# Challenges faced by caregivers of virally non-suppressed children on the intensive adherence counselling program in Uganda: a qualitative study

**DOI:** 10.1186/s12913-019-3963-y

**Published:** 2019-03-07

**Authors:** Esther Nasuuna, Joanita Kigozi, Patience A. Muwanguzi, Joyce Babirye, Laura Kiwala, Alex Muganzi, Nelson Sewankambo, Damalie Nakanjako

**Affiliations:** 1 Infectious Diseases Institute, College of Health Sciences, Makerere University, P.O. Box 22418, Kampala, Uganda; 2 Department of Medicine, College of Health Sciences, Makerere University, P.O. Box 7072, Kampala, Uganda; 3 Department of Nursing, College of Health Sciences, Makerere University, P.O. Box 22418, Kampala, Uganda

**Keywords:** Caregivers, Adherence counselling, Paediatric HIV, Adolescent, Viral suppression

## Abstract

**Background:**

Of the estimated 130,000 children living with HIV in Uganda, 47% are receiving ART. Only 39.3% have suppressed HIV-1 viral load to levels below 50 copies per ml. Caregivers are key drivers of adherence to achieve viral suppression in children. We investigated the challenges and potential support required by caregivers of ART-treated children.

**Methods:**

A qualitative study was conducted within the Infectious Diseases Institute paediatric ART program in Kampala and Hoima districts. Caregivers of children with viral loads above 1000 copies were purposively sampled and engaged in five focus group discussions (FGD). The FGD guide highlighted questions on challenges that caregivers face and the kind of support they required to improve children’s ART adherence. Thematic analysis using the inductive approach was used. All the transcripts were read, coded and emergent themes determined.

**Results:**

Overall, 37 caregivers participated in five FGD, of whom 29 (78%) were female, 28 (76%) were HIV-infected and 25 (68%) were biological parents of the children. The elicited challenges were either in failure to attend the counselling sessions or in supporting adherence to medication. Individual and health system challenges such as competing priorities, logistics, poor quality of counselling and lack of reminders prevented attendance at counselling sessions. Five themes emerged as challenges to supporting adherence: i) environmental (school activities, working away from home), ii) personal (non-disclosure, stigma), iii) psychological (guilt), iv) financial (lack of food and transport) and v) child-related (fatigue and peer influence). Three major themes emerged for the support that caregivers needed namely: a) health system reforms (clinic appointments outside school hours, minimize ART drug stock outs and improve quality of counselling), b) psychosocial support (support with disclosure of HIV status to children and their families, more frequent peer support groups and parenting classes) and c) economic empowerment (training in vocational skills, school fees support and opportunities to initiate income generating activities).

**Discussion and conclusion:**

To achieve viral suppression, ART programs require targeted efforts to provide specific health facility requirements, psychological and economic needs of ART-treated children and their caregivers. Integration of HIV treatment with programs for orphans and vulnerable children may improve viral suppression rates.

## Background

Up to 2.1 million children were living with HIV globally in 2016, 90% of these were living in Sub-Saharan Africa [1, 2]. The increase in Anti-Retroviral Therapy (ART) coverage among children has led to reduction in early mortality, morbidity and improved the quality of life [2–6]. However, challenges to adherence hinder effectiveness of ART [7]. Children have been known to have lower adherence to ART and subsequently lower proportions with viral suppression than ART-treated adults [8, 9].

Uganda has an estimated 130,000 children living with HIV and 47% of them are currently on ART [10]. According to the most recent Uganda Population Health Indicator Survey, viral suppression among children aged 14 years and below is 39.3% [11]. It is important that children are supported to achieve protracted viral suppression during the lifelong ART in order to benefit from it [12]. Poor adherence is one of the commonest causes of viral non-suppression [4, 9, 13, 14]. Adherence is adversely affected by psychosocial factors. Children dealing with the most psychosocial issues have been shown to have the worst adherence to ART [15, 16]. Previous studies have shown non-disclosure, dependence on a caregiver and stigma as some of the reasons for non-adherence [17, 18]. Caregivers play a major role in ensuring that HIV infected children adhere to their medication and attain viral suppression [19]. Caregivers, however, face a multitude of challenges in taking care of HIV positive children; these include depression, lack of food, protracted time off work, loss of employment, stigma from the community, lack of family support and anxiety about death [20–24].

The World Health Organization (WHO) recommends provision of adherence interventions for both adults and children that have a detectable viral load. This has been shown to improve viral suppression by up to 70% [6, 25]. Uganda as a country adopted these recommendations in 2015 and implemented the intensive adherence counselling (IAC) program. IAC offers at least three adherence counselling sessions to viral non-suppressed children after at least six months of ART. Our recent evaluation of the IAC program among children showed poor compliance to the program guidelines and a low re-suppression rate of only 23% among those children that completed the recommended three IAC sessions that were conducted over a three to six month period [26].

With the low viral suppression rates observed after the first year of implementation of the IAC intervention for non-suppression children in Uganda, there is a need to document challenges that caregivers and children face, to inform the development of targeted feasible solutions to improve ART adherence and other treatment outcomes.

## Methods

### Study design and setting

An exploratory qualitative study was conducted at five high patient volume clinics in Kampala and Hoima districts of Uganda. The clinics are supported by the Infectious Diseases Institute (IDI) and are currently implementing the intensified adherence counselling program. This study was informed by a quantitative evaluation of the IAC program which has been described in detail previously [26]. Each participant received a transport refund and simple refreshments after the discussion.

### Participant selection

Purposive sampling was used to recruit caregivers of virally non-suppressed children into the study. These were either biological parents or guardians of children who had gone through the IAC program, but still had a detectable viral load. Participants were invited by phone to participate in the study, those who accepted were requested to come to the facility on specific days where written informed consent to participate in the study including the FGD was obtained. The only respondent below 18 was an emancipated minor as the head of the household and was able to give consent on her own behalf. Ethical approval was sought from the School of Medicine Research and Ethics committee and the Uganda National Council of Science and Technology.

### Data collection

Focus group discussions (FGDs) were held with the caregivers at the facility in a secluded comfortable area. Each FGD consisted of seven to eight [7, 8] people and lasted an average of one hour. The lead researcher (EN) facilitated all the focus groups and a note taker audio recorded the proceedings. A guide with open ended questions and an allowance for probing questions, was used to moderate the discussion. The FGD guide had questions on challenges caregivers faced in attending and completing the sessions in the IAC program, challenges faced in trying to improve adherence for the children, and the support caregivers needed to provide better help for the children to achieve viral suppression. The discussions were conducted in English and the local language and transcribed verbatim immediately after each session. A translator fluent in both English and the local language did the translations into English. EN also fluent in both languages, checked for accuracy of the translations.

### Data analysis

The focus group transcripts were analyzed manually using inductive content analysis. Initially, EN verified the transcription by re-reading the transcribed data while listening to the audio recordings. Four analysts (EN, JK, JB, and LK) reviewed all the transcripts separately then jointly to achieve consensus on the codes emerging from the text. Excerpts of the transcripts were highlighted that spoke to the emerging themes, which included health system, health worker, and individual challenges. When all the transcripts were carefully and exhaustively analyzed, similar codes were grouped and emerging themes determined. EN then organized the content to understand the challenges of the caregivers in supporting children through the IAC program. The data was re-analyzed multiple times to ensure that the explanations were supported by the data. The findings were shared with selected participants to confirm that their message was well represented and to ensure credibility and trustworthiness. These findings were also compared with results from similar studies [15, 17, 27].

## Results

### Characteristics of the study participants

Five FGDs were conducted with 37 participants. 29 were female, 28 were HIV positive, 25 were biological parents of the children, 9 were relatives and 3 were well-wishers (strangers who had taken-in the desolate children). Three of the FGDs were held in an urban setting (Kampala) while the two were in a rural setting (Hoima). See Table 1 for the details of the participants of the focus groups.

**Table 1:** Characteristics of the caregivers that participated in the focus group discussions

Characteristic	Frequency (N)
Age in years	
15 to 25	4
26 to 34	10
35 to 49	16
> 50	7
Sex	
Female	29
Male	8
HIV Status	
HIV Positive	28
HIV negative	9
Relationship to Child	
Biological parents	25
Related to child	9
Well wishers	3
Place of residence	
Urban	21
Rural	16
Directly Observe Medication	
Yes	32
No	4
Lives with the Child	
Yes	35
No	2
Occupation	
Peasant Farmers	12
Petty Trade	14
Housewife	7
Professional	2
Students	2

### Challenges that caregivers face in supporting virally non suppressed children in the IAC program achieve viral suppression

The elicited challenges were in two main areas; supporting the children to attend and complete the IAC program and supporting them to improve adherence to the ART medications.

#### Challenges faced by caregivers in supporting the children to attend and complete the sessions in the IAC program

Caregivers shared that they face multiple challenges that impede their ability to attend or complete the IAC sessions as stipulated in the IAC program. These challenges were either due to the caregiver or the health workers/system. See Table 2.

**Table 2:** Challenges to attendance of Intensive adherence Counselling sessions, as perceived by the caregivers

Theme	Subtheme	Number of respondents^a^
Individual challenges	Fear of death of the child	*5/37*
	Competing Priorities	20/37
	Discouragement	4/37
	No perceived value of sessions	10/37
	Logistics	20/37
Health System Issues	Poor quality of counselling	15/37
	Long queues at the counsellor’s station	18/37
	Unfavourable appointments for school going children	23/37
	Lack of reminders to the caregivers	*6/37*
	Early closure of counselling services at the facility	9/37

^a^Some participants gave multiple responses

The caregiver challenges were mainly: fear of death of the child since they equated high viral load to imminent death; competing priorities such as employment or looking for food; discouragement at failing to achieve viral suppression despite concerted effort; no added value of attending the clinic since they had enough drugs at home and the logistics that accompany bringing a child to the facility such as transport and food.

*I usually have fear. Fear that this time round, my child might die. Then when I interact with other mothers, they tell me that this situation can actually be overcome. Then I gain the courage to go and see the counsellor. The biggest problem is fear.*

*Sometimes you find that we are not self-employed. We have to go out there and look for the children’s school fees, at the same time we have to bring the children to the health facility and you have to seek for a whole day-off from your bosses.*

The health system challenges included: long queues at the facility, poor quality of counselling that was not seen as beneficial, poor scheduling of appointments for school going children, lack of reminders from the health workers and early closure of services at the health facility.

*The reason I avoid going to the counsellor is she is always asking the same question why, why, why, so you say, am I going to go back and she asks me the same thing? She makes you feel like you are doing nothing yet you are doing your level best to ensure your child gets better.*

*When you arrive at the health facility late, they tell you that you have come late when the counselors have already left. So they tell you that they are going to note in your file that when you come for the next appointment, you have to see the counselor first.*

#### Challenges faced by caregivers in supporting the children to improve adherence to the ART medication

**Fig. 1:**
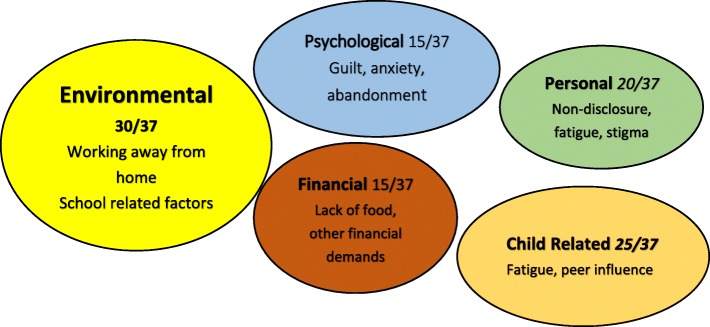
Challenges to improvement in adherence for the children, as expressed by caretakers

A major cornerstone of the IAC program is that it should lead to an improvement in the adherence of the children. However, caregivers expressed some challenges that they face in trying to help the children improve adherence to treatment. Five major themes emerged as the challenges to improvement in adherence. See Fig. 1.

##### The environmental factors

These were related to caregivers working away from home, school related activities that keep children away from home for prolonged periods of time and drug factors such as the drugs being too bitter, too many and the dosing too frequent.

*When we are home with the children, we take the responsibility to ensure that they swallow their drugs. If we are not home, the children sometimes use this opportunity to miss taking their drugs. They at times throw the drugs away and lie to us that they swallowed them.*

*Sometimes my son goes on school trips and comes back after 9 pm yet his evening dose is at 7 pm. This affects him.*

*The children’s drugs are so bitter. One time I was tempted to taste my baby’s syrup and I was shocked to find that it was so bitter. I wondered how my daughter felt every time I gave it to her. I then understood why she sometimes refused to breastfeed or to even take her breakfast after taking the drugs.*

##### The financial factors

Lack of food and infrequent meals prevented children from taking drugs, multiple financial demands on the caregivers led to their inability to give the level of care that they should to their children.

*The counsellors always advise us to never give the children drugs on empty stomachs. However, I usually find myself in a position whereby it is time for the child to take the drugs but I have no food to give them. Sometimes the food is just not yet ready. Therefore, I wait until it is ready, feed them and then give them the drugs even though it is already past time for them to take the drugs.*

*Our mother is seriously struggling and it is usually hard for us to get something to eat. Our brother sometimes goes to school without any money to cater for his meals at school and ends up coming back very hungry in the evening. ….One time, our property owner entered our house and put all of our property outside and then wanted to sleep with me so that we do not have to pay rent anymore but I refused.*

##### Personal factors

Such as non-disclosure of HIV status to the children themselves, spouses and other family members meant that there was limited support for the child; fatigue for both the caregiver and the child in taking daily medication, inability to change practice as the situation could not be resolved and stigma from the community.

*My child always asks why he swallows drugs every day and yet the others do not. I keep telling him that it is because he needs the drugs to stay alive. I cannot tell him the entire truth because the counselors told me that there is a particular age at which I am supposed to disclose to him.*

*….my child started on ARVs when he was 6 weeks old. He is now 12 years old, I have struggled to get him to this point. He should be able to ensure that he takes his drugs by himself now.*

*Much as we attend the counselling sessions, the truth is that we the caregivers do not always put into practice what we discussed with the counsellor during the session… but just continue with what we were doing before.*

… t*hey say that they fear taking the drugs while school because their colleagues always talk about them or laugh at them or even ask them why they are taking the pills.*

##### Psychological challenges

These were maternal guilt at passing on HIV to the children, life uncertainty for the children, lack of family support and abandonment of the caregiver.

*I used to insist that my 12-year-old son takes his medicine despite being tired of the drugs. One day he told me, “Mother you knew you were HIV positive when you were pregnant and you refused to take the drugs, now you infected me and are forcing me to take the drugs.” That was the last day that I ever forced him to take drugs.*

*Sometimes I look at my child and I ask myself, will my child grow? Will my child become someone? Can he travel the world? I hear they do not give visas to [HIV] positive people. Will he attend good schools? Then I feel sad.*

*I have three children, all [HIV] infected, I am the mother, father, aunty, uncle and grandparent. My family rejected them when they discovered they were positive. I have to fulfil all their needs.*

##### Child factors

Such as treatment fatigue and peer influence affect the child and hinder complete adherence to medication.

*For me, my child has started refusing the drugs. She says that given the period for she has been taking the drugs, she is tired of them; she asks me why her heart condition will not heal. Then I tell her to swallow her drugs or else she will die. She then says that if her heart condition will not heal she will then stop taking the drugs. She wishes that she would die.*

*For me, my child is a big man now, he cannot be forced to take his drugs, I can only try but he doesn’t always listen. He has a group [peers] that he listens to more than me.*

### Support that the caregivers would like to receive in order to help the children suppress their HIV viral load

**Fig. 2:**
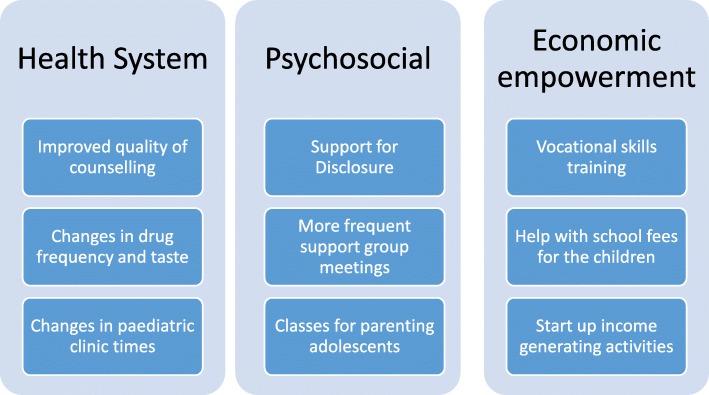
Showing the changes that caregivers would like to see to improve adherence for the children

The caregivers were asked about the kind of support that they would need in order to help the children to improve adherence and suppress their HIV viral loads. The requested support was in three main ways; psychosocial, financial and health system reforms. See Fig. 2.

For psychosocial support, the caregivers observed that the children return encouraged and rejuvenated every time they meet their peers in support group meetings. The children feel less isolated and are encouraged by the fact that they are not alone in the struggle. They asked that the health workers create more opportunities for the children to meet and encourage each other. They should also meet those that are adhering well to encourage them to improve.

*….the various meetings help the children to understand more of their status and what to do, they get motivated to take their drugs after these meetings.*

They also requested health worker support in disclosing the HIV status to the children, their spouses and to other family members especially the ones that oversee care of the children. This will ensure that the children know why they are taking drugs and will hopefully take them better. The partners will be more supportive and start assisting with the daily needs of the children.

*The health workers should come home with us and help us disclose to our partners and families. This will make it much easier on us and get us the help we need from them. We do not know where to start with telling them we and the children are HIV positive.*

For the health system factors, the quality of counselling was an area that was highlighted frequently. Caregivers wanted more frequent counselling that is tailored to the children and delivered in a child friendly way. The children need more time with the counsellors to address their challenges. In these counselling sessions, they should cover the benefits of taking ARVs very well. They asked that the counsellors should be very friendly with the children so that they open up to them.

*The counsellors need to ensure that they create a bond between themselves and the children. This will make it easier for the children to go to the counsellors whenever they are required to.*

Caregivers also indicated that they have a role to play in supporting the children through both physical and emotional support. In physical support they discussed that they should find more ways to stay with the child at home and avoid leaving them with other people so much of the time. They should accompany children to the facility and supervise the medication. Mothers of the adolescents need to stop leaving care of the adolescents to the adolescents. In emotional support, they indicated that these children need special care, comfort, and love in order to take their drugs very well. The caregivers should be friendly to the child, avoid being harsh and always comfort the child.

*We the caregivers need to accompany our children to the health facilities for their appointments. The children need parental support. It should not only be about providing them with transport to bring them to the facility, asking if they swallowed their drugs and punishing them if they did not.*

The caregivers felt that if they were financially more stable, they could take better care of their children despite the lack of support from the fathers of the children. They suggested training them in skills that can enable them to make money, setting up income generating activities for the stay home mothers and help with paying school fees for their children since it takes up most of their income leaving little for food and other necessities.

*There should be some income generating projects started up here at the health center. We can engage in some of these activities when we come here as we wait in the queue since some of us are stay at home mothers. Earning something through these projects could help lift some weight off of our shoulders.*

Lastly, the caregivers called for changes in the drugs. They find it very difficult to give twice a day dosing for multiple pills. They proposed having a long acting injection that you give the child once every three months and avoid oral dosing daily.

*There should be a way of reducing the large pills to smaller ones so that they become more appealing to the children. The sight of six big tablets is so discouraging for a child. The thought of having to take them twice a day worries them a lot.*

## Discussion

In this study, we set out to explore the challenges that caregivers of children that have a detectable viral load in the IAC program face and to determine the kind of support that they would need, to make viral suppression a reality for their children. We found that the challenges that they faced were in two domains: supporting the children to attend and complete the IAC sessions and supporting improvement in adherence. The support that they needed was psychosocial, economic and health system related.

The caregivers had individual challenges such as competing priorities, fear, anxiety, and expenses related to a clinic visit that impeded their ability to attend the IAC sessions. Health worker related challenges such as poor quality of counselling, lack of reminders about the IAC sessions and unfavorable appointments affected their ability to support the children to complete the IAC sessions. There is limited data on the challenges that caregivers face in supporting children to attend and complete the IAC sessions in the program. For this reason, we had no prior studies to compare our findings to.

Five major themes emerged when caregivers were asked about the challenges in helping the children to improve adherence to ART. The environmental challenges emerged strongest with caregivers unable to supervise the children’s medication due to working away from home and unfavorable school activities as the main reasons. These findings are similar to a study done in Nigeria that showed forgetfulness, travelling, and drugs being finished as reasons for non-adherence [28]. Another study done in Kenya showed that pill burden, bitter taste of the pills and school activities affected adherence among adolescents aged 10 to 16 years [29].

Child factors such as peer pressure and fatigue also emerged as challenges to adherence to ART. This was also reported by adolescents in Kenya [29] and is similar to reports from interviews from the adolescents themselves according to two Ugandan studies [30, 31].

Personal factors such as non-disclosure, fatigue, and stigma also emerged. WHO recommends disclosure to all primary school children as a way of aiding adherence to medication and retention in care [32]. Disclosure of HIV status was a major issue raised by the caregivers. This could be disclosure to the child or to other family members including fathers, siblings and the rest of the extended family. Many of the children were not disclosed to and this has been shown to affect adherence and retention in care [31, 33]. A study in Uganda found that children whose primary caregiver was the only one that knew the HIV status were three times more likely to report poor adherence [34]. The caregivers who had disclosed were mostly abandoned by their partners which made others fearful of disclosing. This is similar to a study done in Nigeria that found PMTCT mothers failed to disclose to partners due to fear of divorce and violence [35]. Adherence was observed to be better among children that had been disclosed to [28].

Lack of food and multiple financial demands were also highlighted as challenges to improved adherence for the children. This was also shown in a Ugandan study about the facilitators and barriers to adherence among adolescents [31]. This was very important in that the adolescents in rural Kenya requested for food and transport to the facility as a means to improve adherence [30].

Psychosocial challenges such as guilt, lack of family support and life uncertainty for the children were reported in this study. Guilt from the biological mothers led them to feeling sorry for the child and giving unscheduled drug holidays. This led to poor adherence and subsequently non-viral suppression. This was also found in a Nigerian study that found that children with a caretaker as the biological mother were less likely to be adherent [28]. Mothers who haven’t disclosed to anyone also affected the child getting drugs on time [28].

Caregivers expressed need for psychosocial support, economic empowerment, and health system reforms as enablers for adherence among the children. These findings are similar to those found in a Ugandan study where adolescents themselves called for more support, involvement in income generating activities and caring health workers [31]. Supported disclosure has been shown to improve both adherence and immunologic suppression among adolescents in rural Kenya [36]. Health workers are very instrumental in the delivery of antiretroviral therapy and a review of the issues that perinatally infected adolescents face recommended the training of health workers in how to handle adolescents [37]. This might help them handle non-suppressing children’s issues much better. A study done in Uganda also showed good care from the health care workers as an enabler to good adherence [31].

The challenges that caregivers face are mainly psychosocial and economic in nature. This is in keeping with the situation in Uganda where most of the care of the sick is left to women who are hardly given any support in this role [38, 39]. Due to the high stigma in the community, caregivers of the children are mostly isolated and left to cope alone with the burden of caring [40]. The poverty levels in the community are also very high and more than half the children live in poverty [41]. This means that a lot of caregiver time is spent on finding food daily and other necessities, leaving less time to support children on ART.

### Limitations

One of the limitations from this study is that the findings come from a proxy source rather than the children experiencing the phenomenon being studied, especially the adolescents, this would have given a more holistic understanding of the barriers to staying adherent to medication. However, since they are still defendant on the caregiver, addressing the challenges of the caregivers could potentially lead to suppressed viral load for the adolescents as well. Literature also shows that the issues raised by the caregivers are very similar to the ones raised by the children themselves in various studies around Africa. Since this was a qualitative study, the findings might not be generalizable to other contexts.

## Conclusions

In this study, we found that caregivers face challenges in attending and completing IAC sessions as well as supporting adherence for the children in their care. These challenges need to be addressed by the health care system, HIV programs and from the caregivers themselves if the IAC program is to lead to suppressed viral load for the children. There is an urgent need to start care differentiation for children in order to help them suppress their viral loads by easing the burden that caregivers face. HIV programs needs to re-evaluate the implementation of the IAC program to incorporate more support for the caregivers as identified in this study.

## Abbreviations

ART: Anti-Retroviral therapy
FGD: Focus Group Discussions
HIV: Human Immunodeficiency Virus
IAC: Intensive Adherence Counselling
IDI: Infectious Diseases Institute
WHO: World Health Organisation
